# Solvent modulation in peptide sub-microfibers obtained by solution blow spinning

**DOI:** 10.3389/fchem.2022.1054347

**Published:** 2022-12-06

**Authors:** Ana Margarida Gonçalves Carvalho Dias, Cícero Cena, Viviane Lutz-Bueno, Raffaele Mezzenga, Ana Marques, Isabel Ferreira, Ana Cecília Afonso Roque

**Affiliations:** ^1^ Associate Laboratory i4HB, Chemistry Department, NOVA School of Science and Technology, Institute for Health and Bioeconomy, Caparica, Portugal; ^2^ UCIBIO—Applied Molecular Biosciences Unit, Department of Chemistry, NOVA School of Science and Technology, NOVA University Lisbon, Caparica, Portugal; ^3^ UFMS—Federal University of Mato Grosso do Sul, Campo Grande, Brazil; ^4^ Department of Health Sciences and Technology, ETH Zürich, Zürich, Switzerland; ^5^ Paul Scherrer Institute, Villigen PSI, Switzerland; ^6^ i3N, Materials Department, School of Science and Technology, NOVA University Lisbon, Caparica, Portugal; ^7^ Physics Department, Faculty of Sciences, University of Lisbon, Lisbon, Portugal

**Keywords:** peptide-based fibers, solution blow-spinning, polymeric fibers, solvent modulation, self-assembly

## Abstract

Peptides possess high chemical diversity at the amino acid sequence level, which further translates into versatile functions. Peptides with self-assembling properties can be processed into diverse formats giving rise to bio-based materials. Peptide-based spun fibers are an interesting format due to high surface-area and versatility, though the field is still in its infancy due to the challenges in applying the synthetic polymer spinning processes to protein fibers to peptides. In this work we show the use of solution blow-spinning to produce peptide fibers. Peptide fiber formation was assisted by the polymer poly (vinyl pyrrolidone) (PVP) in two solvent conditions. Peptide miscibility and further self-assembling propensity in the solvents played a major role in fiber formation. When employing acetic acid as solvent, peptide fibers (0.5 μm) are formed around PVP fibers (0.75 μm), whereas in isopropanol only one type of fibers are formed, consisting of mixed peptide and PVP (1 μm). This report highlights solvent modulation as a mean to obtain different peptide sub-microfibers *via* a single injection nozzle in solution blow spinning. We anticipate this strategy to be applied to other small peptides with self-assembly propensity to obtain multi-functional proteinaceous fibers.

## Graphical Abstract

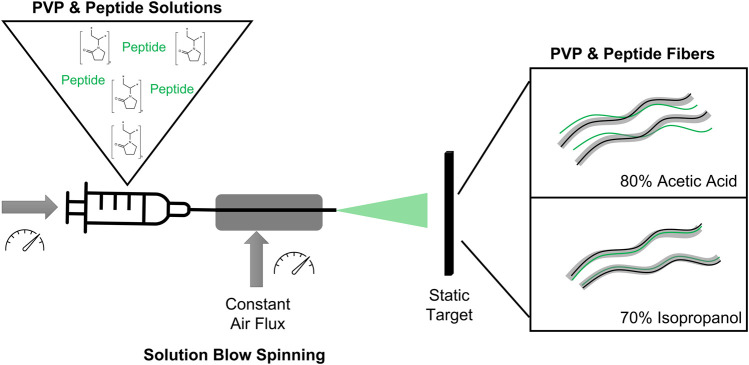

## 1 Introduction

Nature is a source of inspiration to produce innovative, sustainable and biodegradable materials (Katiyar et al., 2021). Peptides with self-assembling properties are interesting molecules to form bio-based materials, as they are composed by a small sequence of amino acids that can be derived from natural sequences, rationally designed or discovered through screening protocols (Berger and Gazit, 2017). Compared to long protein sequences with self-assembly propensity, peptides composed by natural and non-natural amino acids have advantages: they are easy and inexpensive to produce through chemical synthesis in a reliable and scalable manner; they are soluble in water or in non-toxic solvents (Dias et al., 2015; Dias et al., 2016); and they are easily assessed by biophysical and structural studies (Sánchez-Ferrer et al., 2018; Lutz-Bueno et al., 2020). Recently, the field of peptide-based materials has seen great advances due to an increasing understanding of peptide self-assembling principles into higher-ordered structures at the nano-, micro- and even macro-scales (Shaham-Niv et al., 2015; Balasco et al., 2021).

Self-assembled peptides have been traditionally explored to produce hydrogels with various applications in biomedicine and sensing (Amit et al., 2018; Balasco et al., 2021). Recently, it has been acknowledged the untapped opportunities of peptide-based fibers, namely the large surface to aspect ratio associated with large chemical diversity, facile sequence design allow access to different nanoscale architectures, and tunability of optical and electrical properties (Yıldız et al., 2020; Bucci et al., 2021a; Kim et al., 2021). It is known that proteins or small protein precursors (e.g. soya, elastin, spider silk protein or hemoglobin) can be processed by electrospinning to generate protein-fibers for biomaterials and bio-based materials (DeFrates et al., 2018; Yıldız et al., 2020). It has also been demonstrated that peptide sequences composed by hydrophobic amino acids (e.g. Tyrosine, Phenylalanine, Tryptophan), which can naturally self-assemble due to hydrophobic interactions (e.g. π-π stacking), can also form fibers (Balasco et al., 2021). A recent review compiled successful examples of peptide sequences that were spun into fibers through electrospinning (Bucci et al., 2021a). Despite these successes, the main identified challenges to obtain peptide fibers are the low viscosity of peptide solutions, thus requiring high amounts of peptide (10–50% wt/v) to be spun or combination with a polymer (Kim et al., 2021), and the toxicity of solvents used (e.g. hexafluoro-2-propanol (HFIP) and trifluoroacetic acid (TFA)) with a pressing need for more sustainable solutions (Bucci et al., 2021a). Solution blow spinning (SBS) is an alternative method to electrospinning, with advocated main advantages of high production rate and easy implementation (Dadol et al., 2020; Gao et al., 2021). SBS has been used to yield materials from a wide range of natural and synthetic polymers or blends, such as Soya Protein, cellulose, chitosan, poly (vinylidene fluoride), poly (vinyl pyrrolidone) (PVP), poly (L-lactic acid) or poly (vinyl acetate) (Dos Santos et al., 2020). PVP is a low-cost synthetic polymer with low toxicity and good solubility in water or polar solvents. Due its high solubility, PVP can be removed after fibers production by placing them in water (Kurakula and Rao, 2020). Polymer fibers are normally coated with peptides as a post-treatment by physical adsorption or by chemical means (Dart et al., 2019; Butruk-Raszeja et al., 2020; Bucci et al., 2021b; Yao et al., 2023).

In this work, we aim to advance the knowledge on peptide-fiber formation using SBS. We explore a peptide-polymer blend to obtain the desired viscosity at lower peptide concentrations, and by selecting greener solvents (acetic acid and isopropanol (Byrne et al., 2016)) for operation. We used as a proof-of-concept a peptide sequence that self-assembles into higher-ordered structures, identified as the protopeptide YMDMSGYQ, that derives from structural proteins (reflectins) present in cephalopods (Guan et al., 2017). It is postulated that the Tyrosine amino acids in the peptide sequence lead to the self-assembly of the peptide by π-π stacking interactions (Guan et al., 2017). We observed that by changing the solvent we could achieve fibers with different morphologies and diameters. Consequently, we concluded that we can obtain fibrous materials with different properties by solvent modulation.

## 2 Materials and methods

### 2.1 Reagents

The peptide YMDMSGYQ was chemically synthesized (Trifluoroacetic acid free and free terminals) with 97% purity at Genecust (France). Poly (vinyl pyrrolidone) Molecular Weight 360000Da powder, Congo red BioXtra grade, sodium chloride, acetic acid glacial >99% and isopropanol >99% were purchased from Sigma-Aldrich.

### 2.2 Fiber production by solution blow-spinning

Four different solutions (1.6–2.0 ml) were prepared (Supplementary Table S1): PVP AA was composed by PVP in 80:20 (v/v) acetic acid in water; PVP Pep AA was composed by PVP and peptide in 80:20 (v/v) acetic acid in water; PVP Iso was composed by PVP in 70:30 (v/v) isopropanol in water; PVP Pep Iso consisted of PVP and peptide in 70:30 (v/v) isopropanol in water. The solutions were prepared by dissolving PVP (10% wt/v) in the solvent at 40°C under magnetic stirring, after which 100 μl of a peptide solution (Pep AA or Pep Iso at 12% wt/v) in each solvent. The final mixture was composed by PVP (10% wt/v) and peptide (0.75%wt/v). The mixture was incubated for 10 min at 40°C with continuous agitation. A syringe cleaned with the solvent was loaded with the solution and fibers were prepared using a solution blow spinning setup as described elsewhere (Cena et al., 2018). The flow rate used was 0.05 ml/min with an air pressure of 1 MPa. Fibers were projected into a static target with a 50 cm working distance from the nozzle. The fiber spinning was conducted in a room with temperature of 21°C and with relative humidity of 60–70%.

### 2.3 Congo red staining

Solution or fibers produced by SBS (50 μl) were placed in a glass slide, and 20 μl of Congo red solution (20 mg in 10 ml of saturated ethanol solution (2.5 g NaCl with 80% EtOH) (Yakupova et al., 2019)) were added. Any excess of Congo red solution was removed with a lint free paper. The glass slides were observed at the ZEISS Axio Observer Microscope 5, 10, and 40 objectives and under bright field and cross-polarized filters (90°angle).

### 2.4 Atomic force microscopy

All polymer and polymer-peptide solutions (10 μl) were loaded into mica layer and dried overnight in a dust free environment. The surface topography and roughness of viscous solutions were analyzed with the Atomic Force Microscope integrated in the WITec Alpha 300 RAS confocal spectrometer. The cantilever was operated with an Al coated probe in AC mode at 75 kHz and constant load of 2.8 N/m. Lateral and depth resolutions were 1 nm and 0.3 nm, respectively. For all sample’s roughness was determined through the topography maps root mean square height (Sq).

### 2.5 Scanning electron microscopy (SEM)

Scanning electron microscopy (SEM) observations were conducted on a Carl Zeiss AURIGA CrossBeam (FIB-SEM) workstation coupled with energy dispersive X-ray spectroscopy (EDS, Oxford X-Max 150 detector with Aztec software). It was also used TM3030Plus tabletop Microscope from Hitachi. The fibers were previously coated with an Pd conductive film to avoid charge effects.

### 2.6 Attenuated total reflectance–Fourier transform infrared spectroscopy

Fourier-transform infrared spectroscopy (ATR-FTIR) characterization of the fibers. PVP fibers and PVP-Peptide fibers produced in solvents and deuterium water. Spectra were recorded using Spectrum Two with UTAR two adapter from Perkin Elmer. Scans were recorded in absorption mode and as a background air was used. It was used force gauge of 83. In total 25 scans were obtained and averaged with the range from 4000 to 400cm^−1^.

### 2.7 Small-angle X-ray scattering (SAXS) and wide-angle X-ray scattering (WAXS)

The scattering measurements were performed with Rigaku MicroMax-002+ micro-focused beam (4 kW, 45 kV, and 0.88 mA). The copper Kα energy with λe = 1.54 Å was collimated by three pinholes onto a beam size of 700 × 700 μm^2^. A two-dimensional argon-filled Triton detector was used for collecting SAXS patterns, and a Fuji Film BAS-MS 2025 imaging plate for WAXS. The active range q = 0.001–0.200 Å^−1^ was probed for SAXS, and q = 0.2–2.5 Å^−1^ for WAXS. Polymer only and polymer-peptide fibers (PVP AA, PVP Pep AA, PVP Iso and PVP Pep Iso) with diameters between 0.1 and 3 µm were vertically mounted, and measured simultaneously by WAXS and SAXS for 3 h (Lara et al., 2012; Lutz-Bueno et al., 2020).

## 3 Results and discussion

Peptide solutions can be processed into fibers using spinning procedures (Khadka and Haynie, 2012; Dart et al., 2019; Hosseini et al., 2019; Yıldız et al., 2020; Kim et al., 2021). Still, improvements are needed to accommodate the low viscosity of peptide solutions and the low sustainability of solvents used during spinning (Bucci et al., 2021a). We approached these challenges by adding a polymer to the peptide solution to promote fiber formation at lower peptide concentrations (0.75% wt/v). In addition, we attempted the dissolution of the peptide and polymer in environmentally friendly solvents, namely acetic acid and isopropanol, and tested fiber formation by solution blow spinning (SBS).

The peptide sequence YMDMSGYQ was selected as a model peptide for our studies. This octapeptide has two Tyrosine residues in the extremities of the sequence, which are suggested to promote peptide self-assembly and further organization into higher-ordered structures (Guan et al., 2017). Tyrosine residues promote π-π stacking and hydrogen bond interactions due to the tyrosyl side chain, allowing for mixed mode intermolecular interactions (Lee et al., 2019). In fact, we conducted preliminary studies which show that the peptide self-assembly is concentration and time-dependent in aqueous media at low pH, giving rise to hydrogels. The peptide self-assembles into nanofibers as determined by AFM (Supplementary Figure S1A). The adopted secondary structure is likely strongly influenced by stacking of aromatic residues, showing an intense peak at 230 nm in Circular Dichroism (Supplementary Figure S1B), and it is predominantly based on β-sheets, due to an intense peak at 1615 cm^−1^ observed by deconvolution of Attenuated total reflectance–Fourier transform infrared spectroscopy (ATR-FTIR) spectrum in Amide I region (1700 and 1600 cm^−1^) (Supplementary Figure S1C).

**FIGURE 1:**
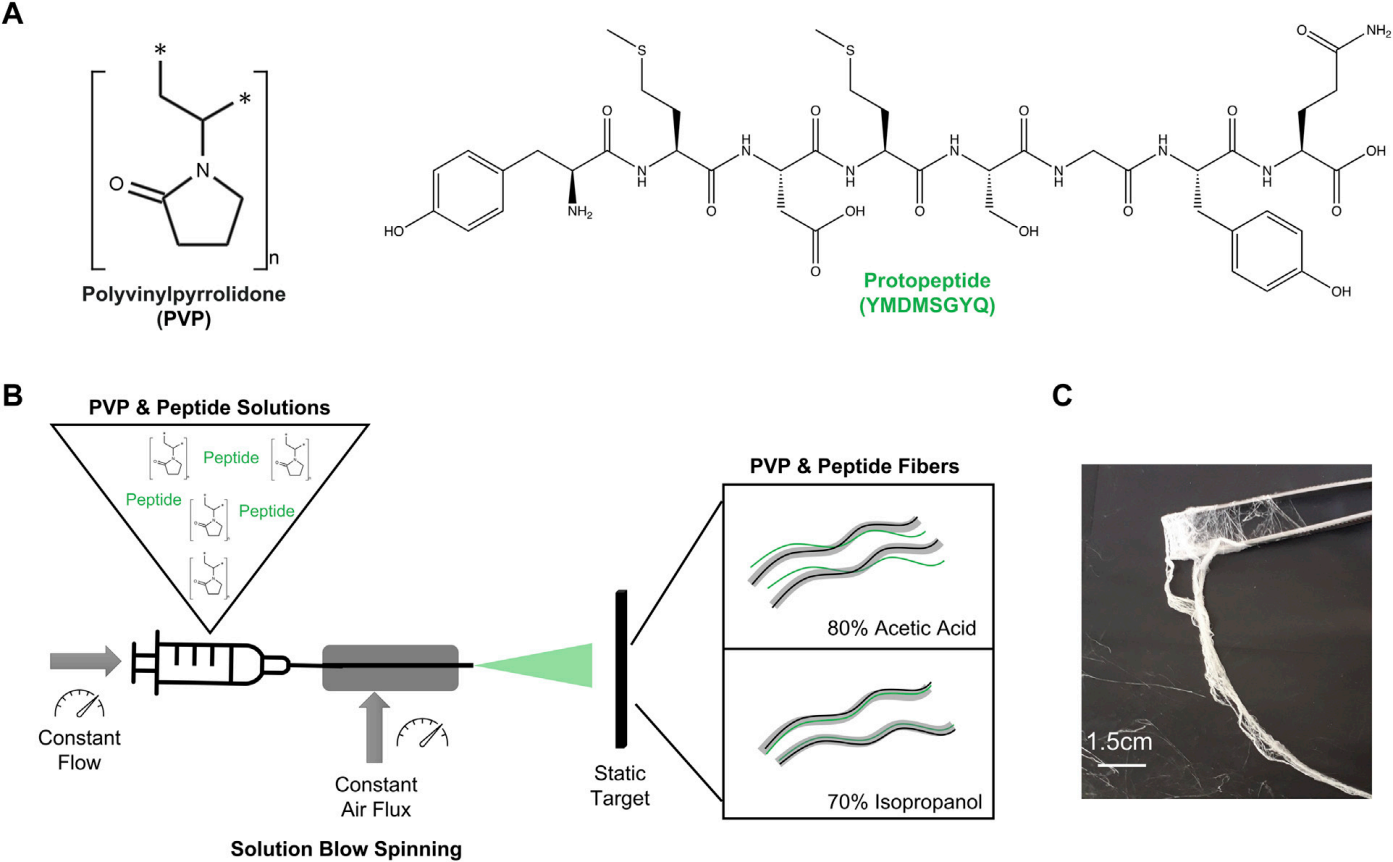
**(A)** Structures of the components used in this work: Polymer—PVP and peptide—Protopeptide; **(B)** Research strategy to produce fibers using solutions with polymer and peptide in two different solvents: 80% acetic acid (80:20 (v/v) acetic acid in water) and 70% Isopropanol (70:30 (v/v) isopropanol in water). The method to spun the solutions into the fibers was solution blow spinning. **(C)** Macroscopic fibers obtained through solution blow spinning.

To increase the viscosity of peptide solutions, we combined the peptide with PVP. PVP is the most used synthetic polymer to produce fibers by solution blow spinning (Dadol et al., 2020; Dos Santos et al., 2020) and it has high solubility in water and polar solvents (Kurakula and Rao, 2020) (Figure 1A). We selected two solvents to dissolve PVP and the peptide: 1) 80:20 (v/v) acetic acid in water, and 2) 70:30 (v/v) isopropanol in water (Figure 1B). These solvents were selected as both are less toxic than the traditional hexafluoro-2-propanol (HFIP) and trifluoroacetic acid (TFA) and present high volatility. Four aqueous solutions were then prepared as starting materials for SBS processing: PVP only (PVP AA), PVP with Peptide (PVP Pep AA) in 80:20 (v/v) acetic acid in water; and PVP only (PVP Iso) and PVP with peptide (PVP Pep Iso) in 70:30 (v/v) isopropanol in water (Supplementary Table S1).

Acetic acid is a polar solvent that mostly contributes as an H-bond acceptor. Isopropanol can establish hydrophobic interactions through the alkyl chain and hydrogen bonds through the H-bond donor capabilities of the alcohol group. PVP has a carbonyl group that is a H-bond acceptor, and it also presents a negative charge at most pH values (isoelectric point (pI) < 3). PVP can form different interactions in acetic acid and isopropanol and it is in fact known to be highly soluble in water, isopropanol and acetic acid (Cena et al., 2018). We observed a proper solubilization of PVP in both solvents (Supplementary Figure S2A). The peptide presents a low pI (pI = 3.7) being positively charged in acetic acid solution (peptide solution with pH 2–3), and neutral to slightly negative in the isopropanol solution (peptide solution with pH 4). Being an octapeptide, it can establish a multitude of interactions, namely hydrophobic and hydrogen bonds through the backbone (both as H-bond donor and acceptor) and through amino acids side chains, such as Tyrosine, Glutamine, Serine and Aspartic acid residues. The peptide was readily dissolved in the acetic acid solution yielding a transparent solution (Pep AA, Supplementary Figure S2A). By adding the peptide to the PVP solution in acetic acid (PVP Pep AA) the transparency of the solution was maintained, but it was possible to observe the formation of peptide aggregates upon Congo red staining, which were less visible in the solution containing only peptide (control) (Supplementary Figure S2B). This indicates that the peptide can self-assemble into ordered structures in the acetic acid solution with PVP polymer. Conversely, the dissolution of peptide in isopropanol resulted in a white flocculated solution with poor solubility, yielding large peptide aggregates with green birefringence when stained with Congo red (Pep Iso, Supplementary Figures S2A,B). However, when the peptide solution was added to the PVP solution in isopropanol (PVP Pep Iso) and incubated at 40°C, a translucid solution was formed suggesting an improvement in peptide solubility in the presence of the polymer. This result was confirmed by the absence of peptide aggregates when staining with Congo Red (Supplementary Figure S2B). In PVP Pep Iso solution we may have predominantly hydrophobic interactions between the peptide (which is neutral to weekly negative) and the polymer chain, which are favored by the presence of isopropanol.

The solutions were further characterized by AFM (Supplementary Figure S3), and it was visible the presence of aggregates in PVP Pep AA, in contrast with PVP Pep Iso, which presents a surface with some texture. Besides, to quantify the roughness in the mica surface it was determined the root mean square height (Sq) for all samples (Supplementary Figure S3C). PVP Pep AA displays the highest Sq among all samples, thus indicating large volumes in the surface of the mica. These large volumes can indicate that there are aggregates of peptide. In solutions of PVP Pep Iso, the opposite occurs with lowest values of roughness. These results agree with the observations of Congo red staining. Thus, in presence of the polymer, the peptide self-assembles into higher-order aggregates in acetic acid and it is in a monomeric format in isopropanol.

**FIGURE 2:**
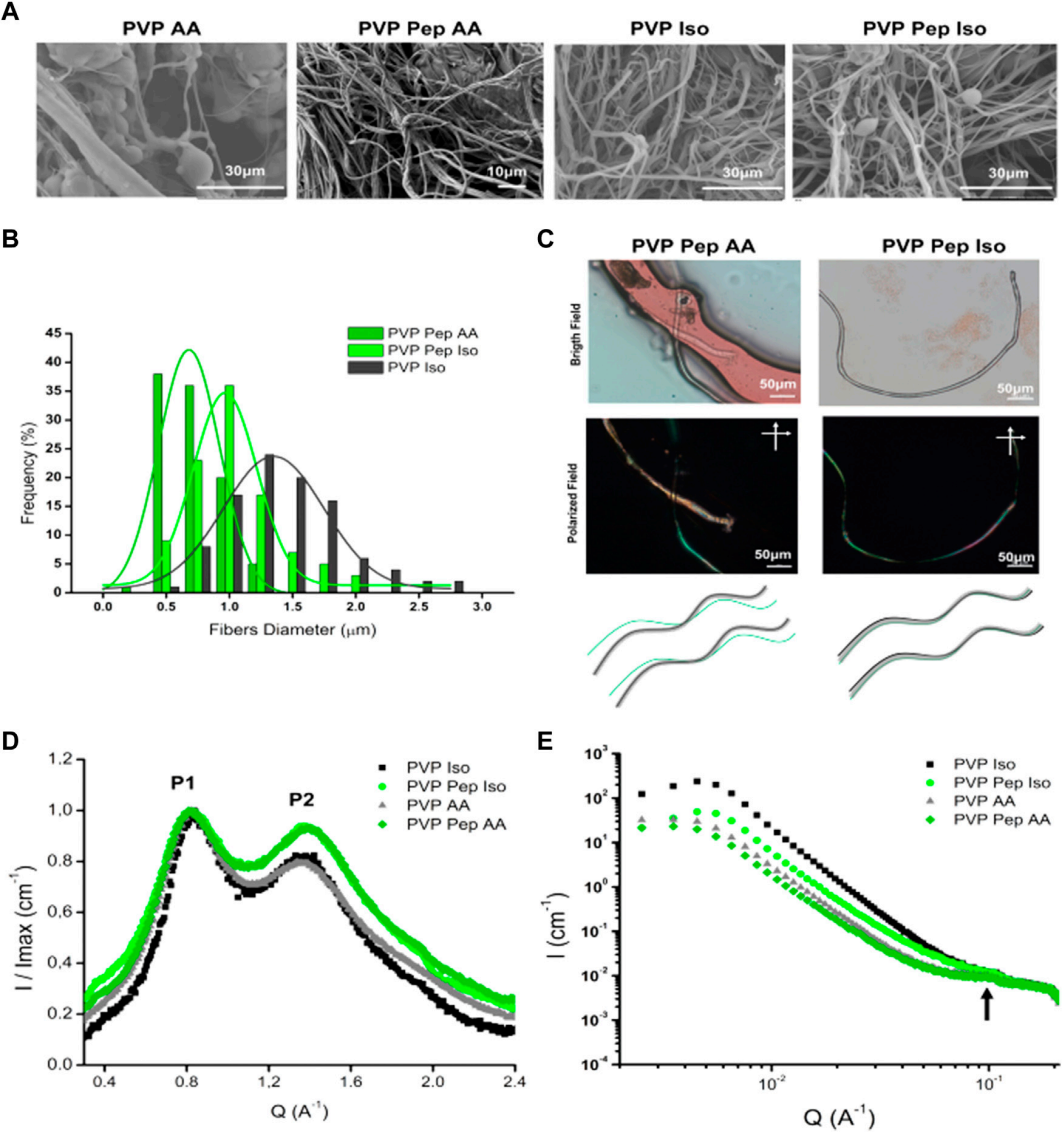
Characterization of polymer and peptide fibers produced by Solution Blow Spinning. **(A)** SEM analysis of fibers produced in 80% Acetic Acid (AA) and 70% isopropanol (Iso). It was observed several defects in PVP AA fibers and all the other samples present fibers without defects. **(B)** Histogram of the fibers diameter for samples: PVP Pep AA, PVP Pep Iso and PVP Iso. **(C)** In the upper panel, microscopic observation of Congo red staining for PVP Pep AA and PVP Pep Iso fibers. In PVP Pep AA, in bright field are observed two fibers: one main fiber in red and a second thinner fiber, but under polarized field (cross polarized at 90°angle) only the thinner fiber presents green birefringence. In PVP Pep Iso, only one fiber is observed in bright field and in polarized field has areas with green birefringence. In the lower panel, schematic of the microscopic observations, for the PVP with peptide fibers obtained in the different solvents: a main fiber of PVP (black), a peptide fiber (green) and a fiber that contain both compounds (grey). **(D)** WAXS, the P1 and P2 identify two main peaks observed in the different fibers. **(E)** SAXS data for the different fibers, the arrow identifies a weak correlation peak.

The solutions were then processed into fibers by solution blow spinning and all yielded fibers (Figure 2). The control solution PVP AA gave rise to fibers with several morphological defects as “dried particles” and beads, whereas PVP Iso originated well-defined fibers (smooth and cylindrical) (Figure 2A; Supplementary Figure S4A). PVP Pep AA also presented well-defined fibers with different diameters and PVP Pep Iso demonstrate a mixture of fibers with morphological defects as “beads” and “dried particles” (Cena et al., 2018). The diameter of the different fibers was measured (except for the PVP AA control due to the high number of defects) and a monomodal fiber diameter distribution was observed (Figure 2B). The PVP Iso fibers showed a wide diameter distribution from 0.50–2.75 µm, centered around 1.34 µm. A narrow fiber diameter distribution was observed for PVP Pep AA, with the diameter centered around 0.68 µm. The PVP Pep Iso fibers showed an intermediary fiber diameter distribution, centered around 0.96 µm, between PVP Iso and PVP Pep AA. Adding the peptide to the PVP solution induces the formation of thinner fibers, probably by reducing the viscosity of the solutions, as verified before (Farias et al., 2020). Despite some variability, all fibers diameters are well in accordance with the data described in the literature for solution blow-spinning (Cena et al., 2018).

A further question arose on how the peptide assembled in the fibers (Figure 2C; Supplementary Figures S4, S5). In the case of PVP Pep AA fibers, we observed the presence of distinct fibers upon Congo red staining–a main fiber and a secondary fiber, which is a thinner entangled fiber. In bright field both fibers stained red, however under cross polarized light only the thinner fiber presented green birefringence. This is typically associated with peptide and protein-based amyloid or amyloid-like aggregates (Shaham-Niv et al., 2015; Yakupova et al., 2019), suggesting that the thinner fiber contains higher amounts of organized peptide structures and the main fiber contains mainly the PVP polymer. In contrast, for PVP Pep Iso only one fiber is visible, and it also presents areas with green birefringence upon Congo red staining, indicating that the peptide is co-organized into higher order structures in the presence of PVP polymer. The fibers were also characterized by ATR-FTIR. When we compared the spectra of fibers with PVP and peptide, we do not see major differences from the control with PVP only. This is due to the low amount of peptide in the mixture, with PVP spectra masking peptide peaks. Control PVP fibers present intense characteristic peaks C=O at 1660cm^−1^ and C-N stretching at 1264cm^−1^ (Cena et al., 2018) (Supplementary Figure S6). The deconvolution of Amide I band was not possible, and we could not use this method to infer on the secondary structure of the peptide within the fibers.

Fibers were also characterized by X-ray scattering in an attempt to understand the assembly of the peptide within the fibers. Figure 2D compares the WAXS signal as integrated curves from the different fibers, after normalizing their intensity by the maximum intensity value. For all fibers independent of the solvent, the WAXS signal has two reflection peaks–P1 ∼ 0.8 Å^-1^ and P2 ∼ 1.35–1.39 Å^-1^. We assign these peaks to the reflections at 0.7 and 1.2 Å^-1^, which are characteristic fingerprints of polymers (Busselez et al., 2012). These results suggest that the peptide signals of a possible amyloid structure, beta-sheets or beta-strands are being masked by the strong PVP signals that occur in the same q-range. Due to the overlap of signals, it is not possible to identify the structure of the peptides within these fibers. However, the main difference between these samples is that PVP Pep fibers exhibit higher intensities for P2 in both solvents. This peak is usually associated with hydrogen bond interactions in amyloids, and it is related to beta-strands. Thus, these results suggest the presence of a compound in the fiber that promotes these interactions. In fact, from previous studies to determine the secondary structure of the peptide by circular dichroism, we identified a strong beta-sheet secondary structure content when the peptide assembles into higher structures (Supplementary Figure S1). The SAXS curves indicated a weak correlation peak at q ∼ 1 A^−1^ (Figure 2E, arrow), which indicates that these fibers have a certain regularity in spacing in the length scale of about 63 Å. From complementary studies with this peptide, we speculate that such dimension of 63 Å could be related to the distance between the peptide’s chains (data not shown).

It should be noted that the formation of peptide-only fibers has been attempted. Peptide solutions in 80:20 (v/v) acetic acid in water were prepared at concentrations 0.75, 3, 5, 7.5, and 15% wt/v and the viscosity measured in a rotary viscosimeter. It was observed a linear correlation between the concentration and viscosity of the solutions until 5%wt/v. For higher concentrations, the viscosity measurements were very unsatisfactory, since as soon as the solution was deposited in a surface, the solvent would evaporate, and the peptide gelate (Table 1). Despite that, the 15% wt/v solution showed the highest viscosity results and closer to the desired viscosity for SBS (0.45–0.5 Pa.S) (Cena et al., 2018). However, when attempting to process the 15% wt/v solution by SBS, the peptide started to gelate as soon as the solvent evaporate in the tip of the nozzle, due to the high concentration. Thus, the peptide in acetic acid self-assembles at concentrations ideal for SBS, but it is difficult to have the perfect balance between peptide concentration, viscosity and gelation control.

**TABLE 1:** Viscosity measurement of peptide solutions in 80:20 (v/v) acetic acid in water using CAP 2000^+^ Viscosimeter (AMETEK Brookfield) following manufacturer instructions.

Peptide concentration (% wt/v)	Viscosity (Pa.S)
0.75	0.15 ± 0.01
3	0.20 ± 0.02
5	0.29 ± 0.02
7.5	0.19 ± 0.04
15	0.34 ± 0.04

**FIGURE 3:**
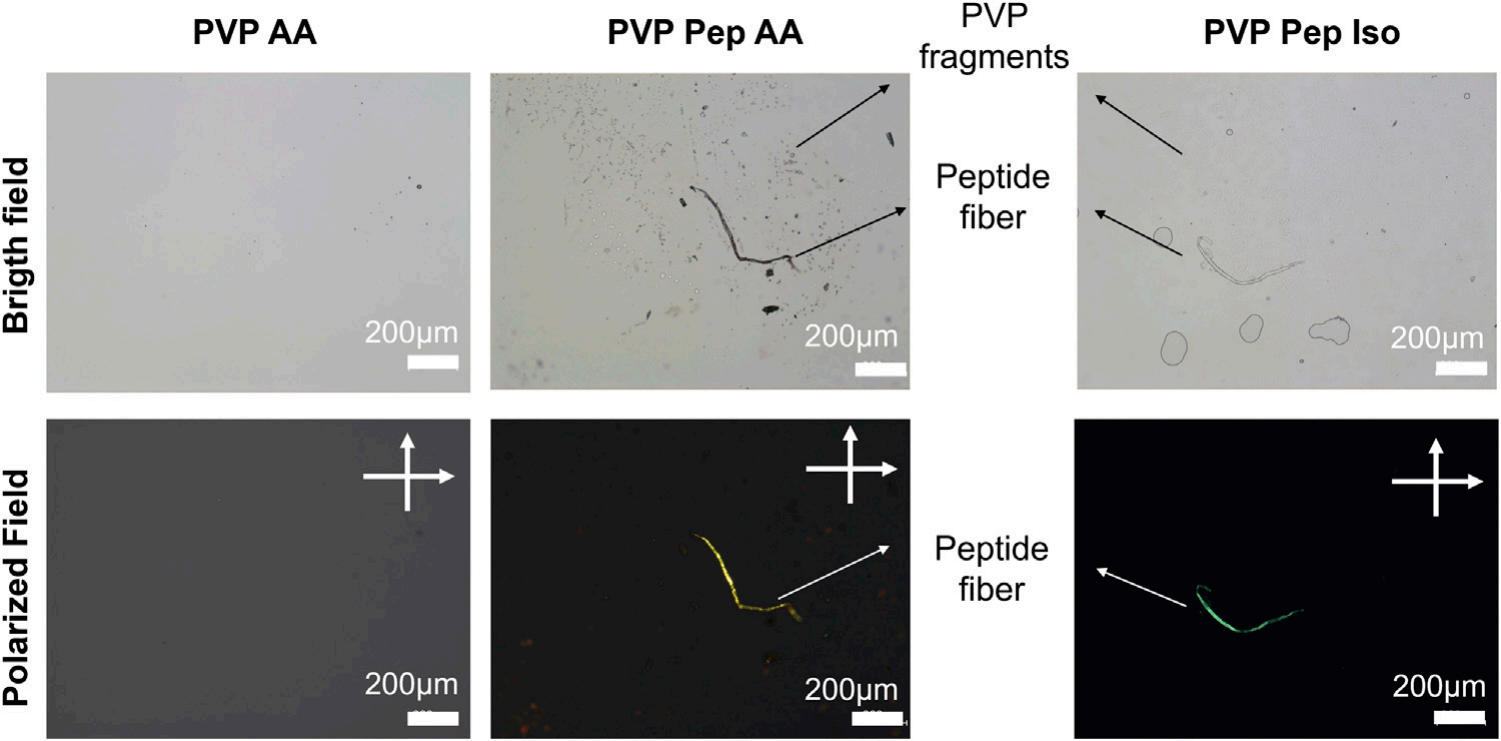
Microscopic observation of PVP and peptide fibers dissolution and staining with Congo Red in bright and polarized field (cross polarized at 90° angle).

Therefore, the use of PVP to co-spin peptide fibers contributes for an ideal viscosity of the peptide solutions. In addition, PVP can be removed after fibers production due to its water solubility. We verified this second advantage by placing PVP Pep AA and PVP Pep Iso fibers in a glass slide which was wet with water to solubilize PVP. In Figure 3 it is possible to observe one fiber with some fragments suggesting that PVP was dissolved, especially visible in the PVP Pep AA sample. This fiber was further stained with Congo red and it presented green birefringence (suggesting the presence of organized peptide assemblies) whereas the small fragments did not.

## 4 Conclusion

We describe a strategy for solvent modulation to obtain different peptide fibers with only one injection nozzle using solution blow spinning. Our results demonstrate that the miscibility of the peptide in different solvents in the presence of PVP induces the production of fibers with distinct morphologies. In particular, the propensity for peptide self-assembly in distinct solvents seems to be the key aspect to induce the formation of PVP-enriched and peptide-enriched fibers (as in the case of acetic acid) or the formation of co-assembled fibers PVP-peptide (in isopropanol). Furthermore, PVP fibers can be dissolved without impairing peptide fiber integrity. This solvent modulation strategy opens a new approach for production of peptides-based fibers for different bioengineering applications.

## Data Availability

The raw data supporting the conclusion of this article will be made available by the authors, without undue reservation.
