# Supplementary material for: Solvent modulation in peptide sub-microfibers obtained by solution blow spinning
Source: Front Chem. 2022 Dec 6;10:1054347. doi: 10.3389/fchem.2022.1054347 (PMC9763608; doi:10.3389/fchem.2022.1054347)
Supplement: Supplementary file 1 [file DataSheet1.docx]

Supplementary Material

**Solvent modulation in peptide sub-microfibers obtained by solution blow spinning**

**Ana Margarida Gonçalves Carvalho Dias ^1,2^*, Cícero Cena ^3^, Viviane Lutz-Bueno ^4,5^, Raffaele Mezzenga^4^, Ana Marques ^6,7^, Isabel Ferreira ^6^, Ana Cecília Afonso Roque ^1,2^***

^1^ Associate Laboratory i4HB - Institute for Health and Bioeconomy, Chemistry Department, NOVA School of Science and Technology, Campus Caparica, 2829-516 Caparica, Portugal;

^2^ UCIBIO – Applied Molecular Biosciences Unit, Department of Chemistry, NOVA School of Science and Technology, NOVA University Lisbon, 2829-516 Caparica, Portugal;

^3^ UFMS – Federal University of Mato Grosso do Sul, Campo Grande, MS, Brazil;

^4^ Department of Health Sciences and Technology, ETH Zürich, 8092, Zürich, Switzerland;

^5^ Paul Scherrer Institute, 5232, Villigen PSI, Switzerland

^6^ i3N, Materials Department, School of Science and Technology, NOVA University Lisbon, 2829-516 Caparica, Portugal;

^7^ Physics Department, Faculty of Sciences, University of Lisbon, 1749-016 Lisbon, Portugal

*** Correspondence:**Corresponding Author
margarida.dias@fct.unl.pt and cecilia.roque@fct.unl.pt

Supplementary Figures


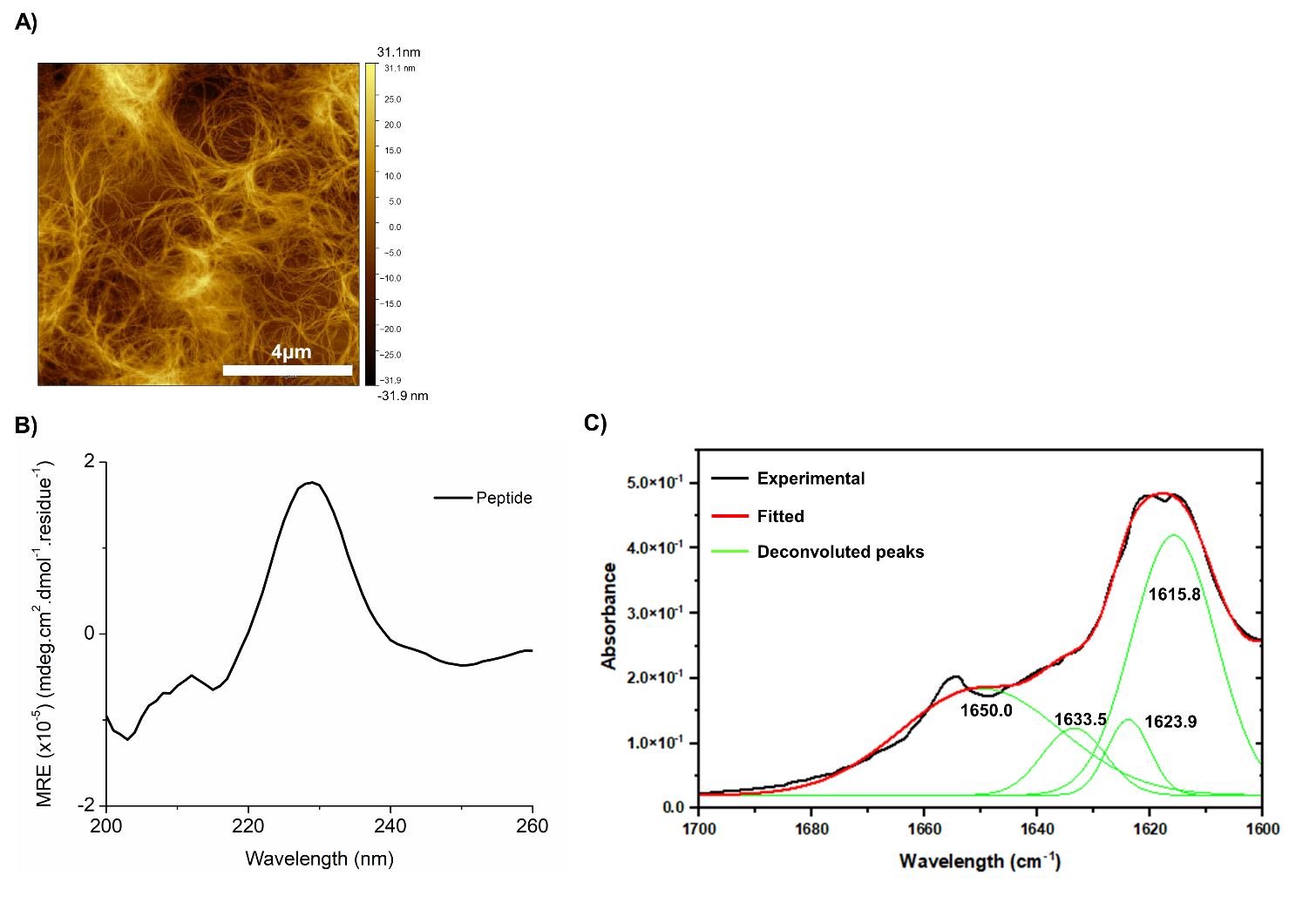


**Figure S1** – Protopeptide self-assembly preliminary studies. A peptide solution (3.6% wt/v in 20 mM Sodium acetate-acetic acid, 100 mM NaCl at pH 4.0) yielded an hydrogel, which was further characterized by Atomic Force Microscopy (AFM) (A) and by Circular Dichroism (CD) (B) and Attenuated total reflectance – Fourier transform infrared spectroscopy (ATR-FTIR) (C). C) ATR-FTIR analysis - for Amide I band deconvolution was used Origin 2021b software with Peak Deconvolution tool (v.1.6) (1–4), assigned to β-sheet structures (54% of the total conformations) and peaks 1633 cm^-1^ and 1650 cm^-1^ assigned to β-turn and random coil conformations.

Table S1 – Solutions prepared in this work

| Sample name | Solution Composition |
| --- | --- |
| PVP AA | 10% wt/v PVP in 80:20 (v/v) acetic acid in water |
| Pep AA | 12% wt/v Peptide in 80:20 (v/v) acetic acid in water |
| PVP Pep AA | 10% wt/v PVP and 0.75%wt/v peptide in 80:20 (v/v) acetic acid in water |
| PVP Iso | 10% wt/v PVP in 70:30 (v/v) isopropanol in water |
| Pep Iso | 12% wt/v Peptide in 70:30 (v/v) isopropanol in water |
| PVP Pep Iso | 10% wt/v PVP and 0.75%wt/v peptide in 70:30 (v/v) isopropanol in water |


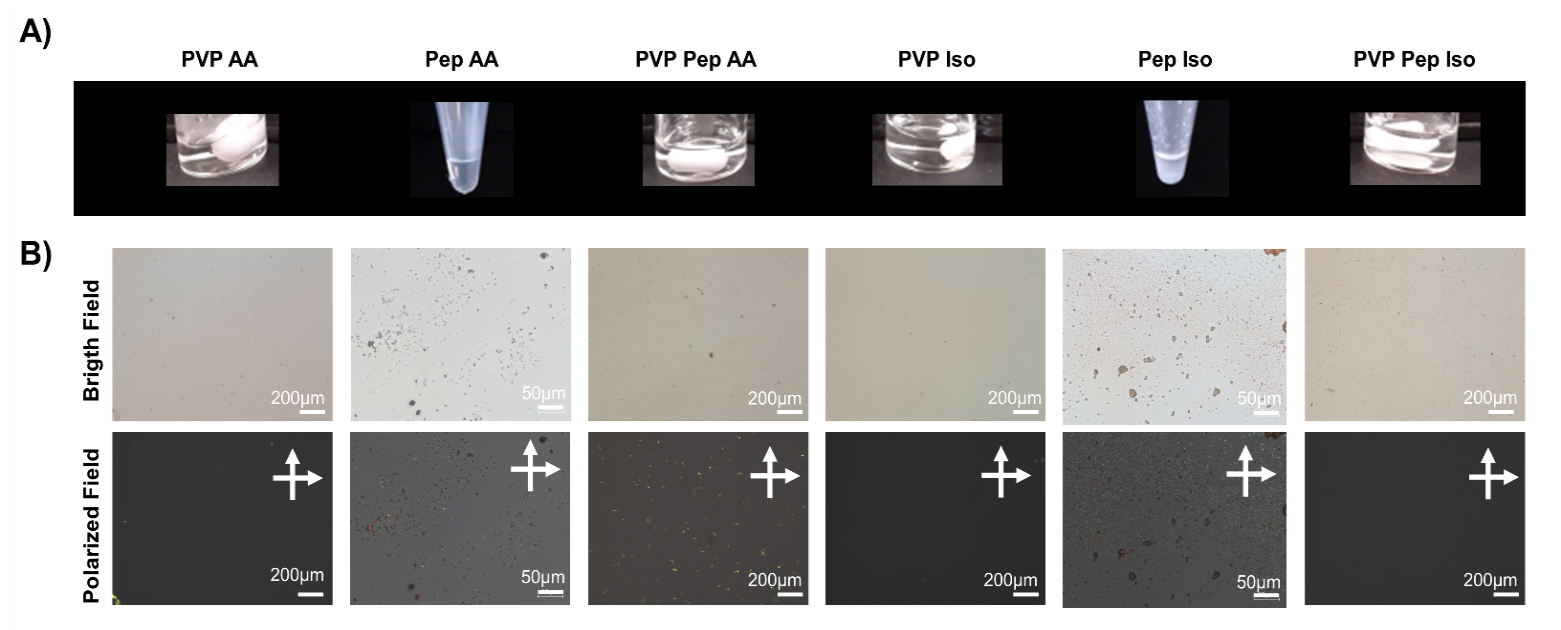


**Figure S2** – A) Photos of the solutions used for solution blow spinning. B) Samples of the solutions were stained with Congo red for the analysis of peptide amyloid like aggregates.


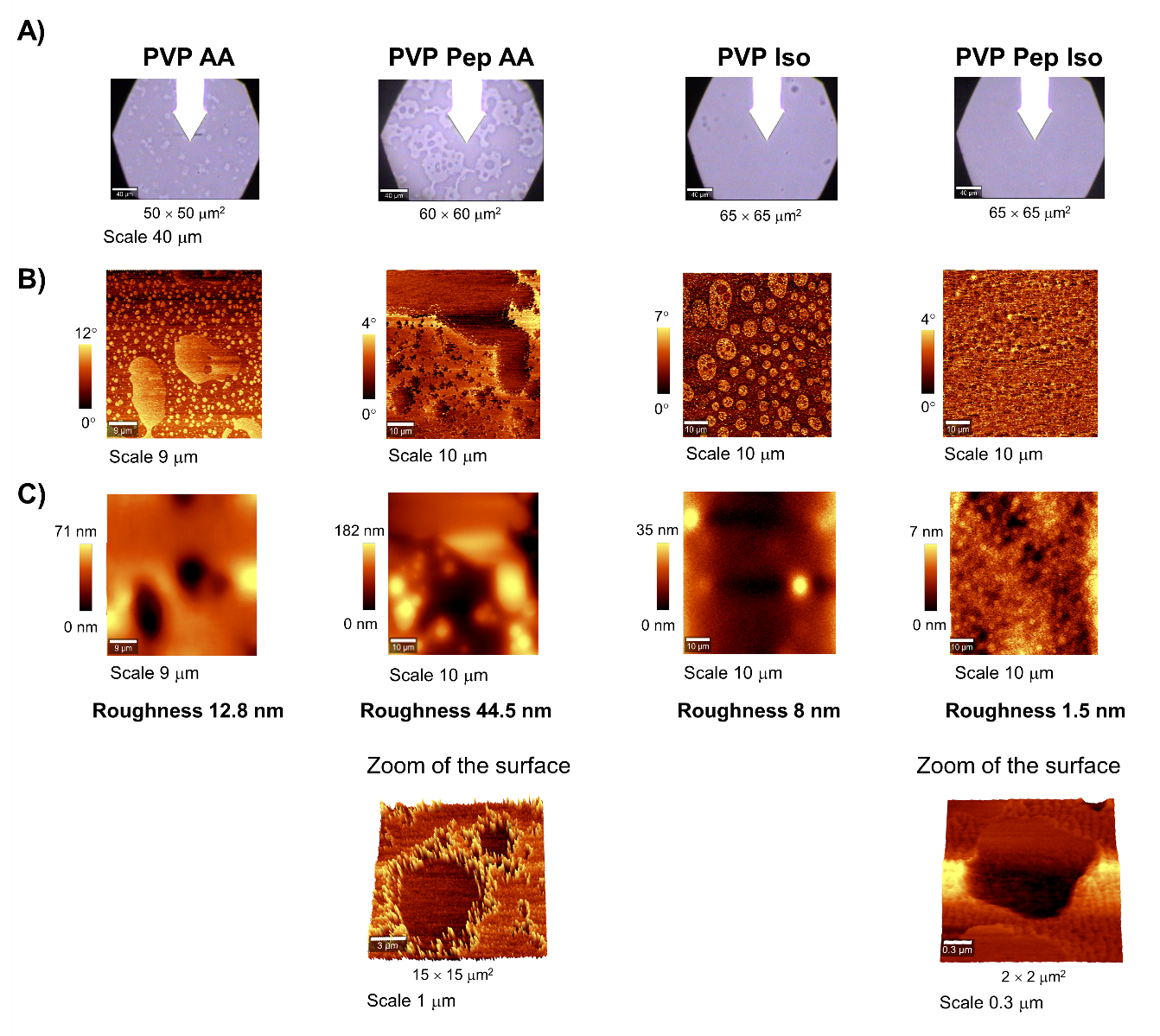


**Figure S3** – AFM Analysis of solution of Polymer and Polymer with Peptide in both solvents: PVP AA; PVP Pep AA; PVP Iso; and PVP Pep Iso. A) Microscopic views of samples deposited in mica (20× optic). B) Phase maps; C) Topography maps. Roughness is given by the root mean square height (Sq) parameter. The PVP Pep AA roughness (44.5 nm) is significantly higher than that of PVP AA (12.8 nm) due to increased surface irregularity, suggesting the formation of aggregates of peptide. Samples of PVP Iso and PVP Pep Iso show considerably lower roughness values (8 nm and 1.5 nm, respectively), which suggests a slight texturization on the surface of the mica.

**
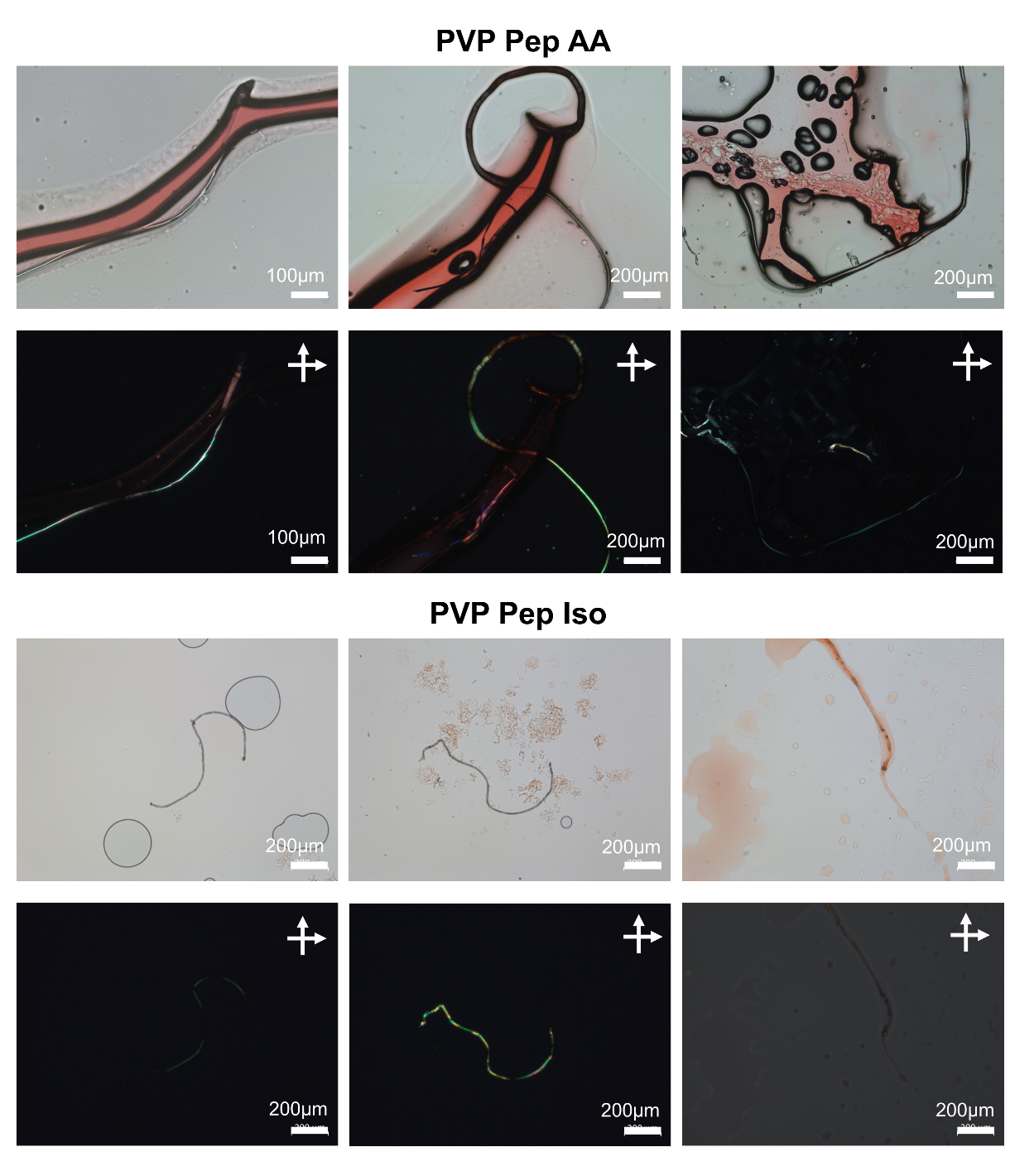
Figure S4** – Fibers produced with PVP and Peptide stained with Congo red for the analysis of peptide amyloid like aggregates.


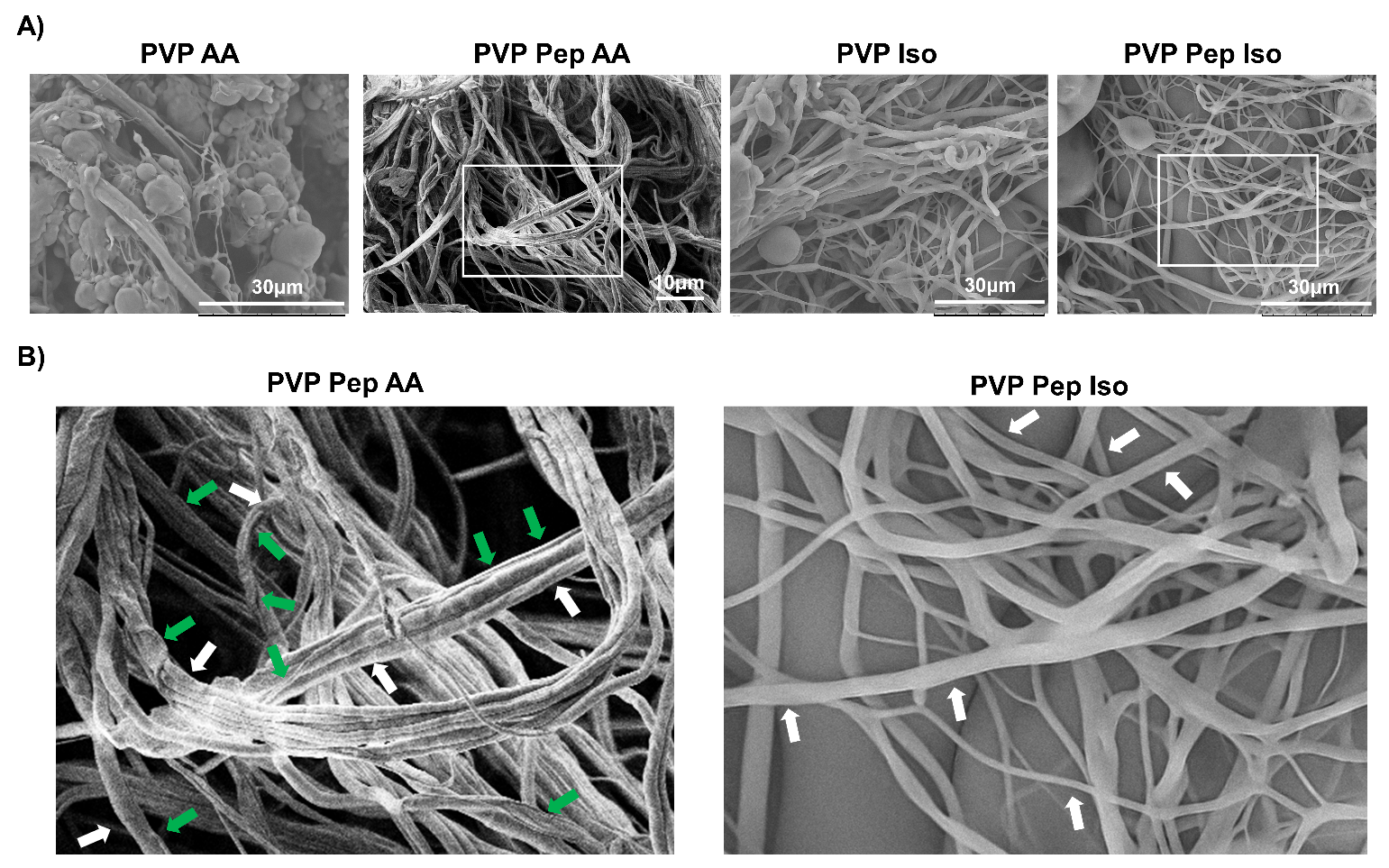


**Figure S5 –** SEM analysis of polymer and peptide fibers produced in 80% Acetic Acid (AA) and 70% isopropanol (Iso). A) Morphological analysis of the different fibers obtained. B) Zoom in section represent the area in the white rectangle in (A) for PVP Pep AA and PVP Pep Iso. Main fiber is identified by white arrows and a secondary fiber is identified by green arrows (peptide).

**
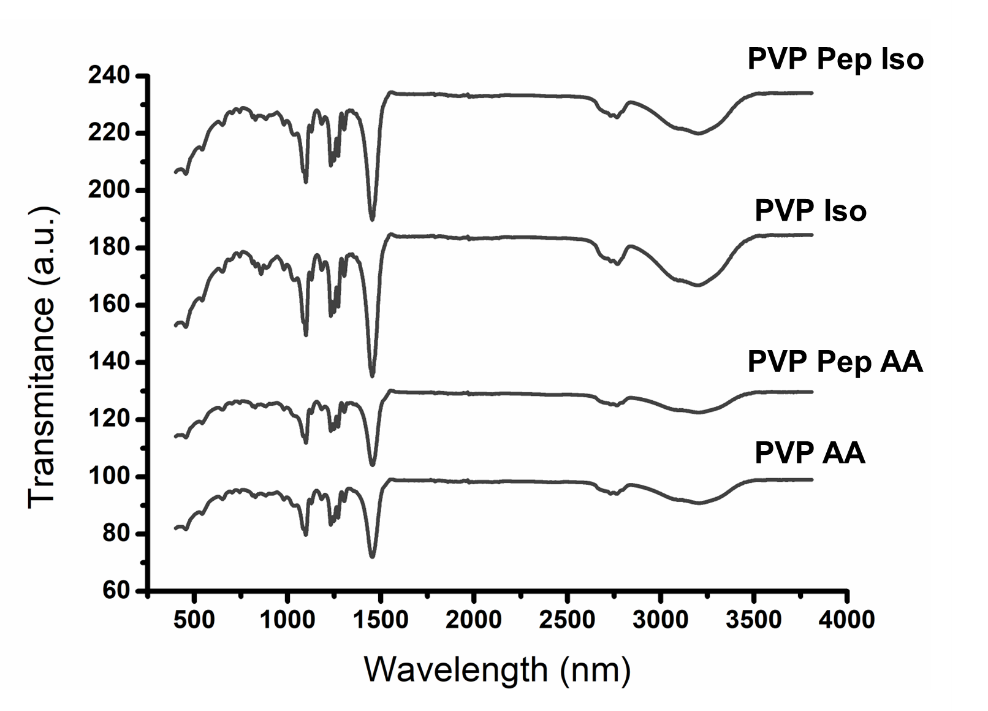
**

**Figure S6 –** Fourier-transform infrared spectroscopy (ATR-FTIR) characterization of the fibers.

**References:**

1. Yang H, Yang S, Kong J, Dong A, Yu S. Obtaining information about protein secondary structures in aqueous solution using Fourier transform IR spectroscopy. Nat Protoc 2015 103 [Internet]. 2015 Feb 5 [cited 2021 Sep 28];10(3):382–96. Available from: https://www.nature.com/articles/nprot.2015.024

2. Vass E, Hollósi M, Besson F, Buchet R. Vibrational Spectroscopic Detection of Beta- and Gamma-Turns in Synthetic and Natural Peptides and Proteins. Chem Rev [Internet]. 2003 [cited 2021 Sep 28];103(5):1917–54. Available from: https://pubs.acs.org/doi/abs/10.1021/cr000100n

3. Batol S, Stefan B, Susana L.A. A, Petra H. Chapter 10 - Secondary Structure Determination by Means of ATR-FTIR Spectroscopy. In: Lacapere J-J, editor. Methods in molecular biology [Internet]. Methods Mol Biol; 2017 [cited 2021 Sep 28]. p. 195–203. Available from: https://pubmed.ncbi.nlm.nih.gov/28755370/

4. Cobb JS, Zai-Rose V, Correia JJ, Janorkar A V. FT-IR Spectroscopic Analysis of the Secondary Structures Present during the Desiccation Induced Aggregation of Elastin-Like Polypeptide on Silica. ACS Omega. 2020;5(14):8403–13.
